# Dr. Frank S. Jackson

**Published:** 1904-02

**Authors:** 


DR. FRANK S. JACKSON, of Dunkirk, died January 1, 1904, aged 52 years. He graduated in medicine at New York University Medical College in 1882, and for many years has been engaged in practice at Dunkirk. He is survived by a widow, father, mother, one brother, and one sister, the latter being Mrs. E. B. Osborne, of Mount Morris. His brother, Robert Jackson, is engaged at the New York Central railroad shops at Depew.

